# Clinical and Pathogenic Characteristics of Lower Respiratory Tract Infection Treated at the Vietnam National Children's Hospital

**DOI:** 10.1155/2020/7931950

**Published:** 2020-03-11

**Authors:** Hien T. Pham, Phuc T. T. Nguyen, Sinh T. Tran, Thuy T. B. Phung

**Affiliations:** ^1^International Outpatient Department of Vietnam National Children's Hospital (VNCH), Hanoi 100000, Vietnam; ^2^Research Biomolecular for Infectious Disease Department of Vietnam National Children's Hospital (VNCH), Hanoi 100000, Vietnam

## Abstract

Lower respiratory tract infections are commonly caused by viruses and cause significant morbidity and mortality among children. Early identification of the pathological agent causing these infections is essential to avoid unnecessary antibiotic use and improve patient management. Multiplex PCR techniques were recently developed to detect multiple viral pathogens using a single PCR reaction. In this study, we identify viral pathogens in children with respiratory infections. We collected 194 nasopharyngeal aspirates from infants (2–24 months old) with lower respiratory tract infections treated at the Vietnam National Children's Hospital between November 2014 and June 2015 and assessed the presence of 16 virus types and subtypes by multiplex PCR using the xTAG Respiratory Viral Panel (RVP) assay. Overall, 73.7% of the samples were positive for at least one virus, and 24.2% corresponded to infections with multiple viruses. The most common viruses were respiratory syncytial virus and enterovirus/rhinovirus. These viruses were more frequent among younger patients (2–5 months old) and caused symptoms similar to those of bronchiolitis and pneumonia. The most common clinical manifestation caused by respiratory tract infection was bronchiolitis. Elevated neutrophils levels were associated with adenovirus infection. Our results showed that the xTAG Respiratory Viral Panel (RVP) can effectively detect multiple viruses causing respiratory infections in children and that the nasopharyngeal aspirates are a good sample choice to detect respiratory viruses in children. Applying this approach in the clinical setting would improve patient management and allow early diagnosis, thus avoiding the unnecessary use of antibiotics.

## 1. Introduction

Acute respiratory infections (ARIs) are common among children worldwide. Although they have similar incidence rate in developed and developing countries, the mortality rate is higher in developing countries [1–3]. ARIs are responsible for approximately 20% of all deaths in children under 5 years in 2006; about 70% of these deaths occur in sub-Saharan Africa and the southern regions of Asia. Viruses are the main causative agents of respiratory infection, but bacteria, fungi, and parasites can cause them as well. The leading ARI-causing viruses are rhinovirus and respiratory syncytial virus (RSV) [4], with RSV affecting mainly infants under 1 year of age [5, 6].

Viruses primarily infect and replicate in the airway epithelium, causing injuries in the proximal (conducting) and distal airways (alveoli and parenchyma), where gas exchange occurs. Viral infection can have multiple clinical manifestations, such as pneumonia, defined as an inflammation of the lung parenchyma. This condition is often associated with visible changes on chest X-rays, CT scanning, or gallium scanning and with abnormalities in alveolar gas exchange. The presentations of viral pneumonia vary considerably depending on the age and immunological competence of the host, as well as the viral pathogen. Viral pneumonia is an important cause of morbidity and mortality in immune compromised individuals and children [7]. The clinical presentation depends on the specific causative agent but typically includes fever and lower respiratory tract symptoms, such as tachypnoea, nonproductive cough, wheeze, and increased breath sound [7, 8].

Inappropriate antibiotic use during acute respiratory infection has led to an increase in antimicrobial resistance [9]. Therefore, early detection of the pathogen causing respiratory infection may prevent antibiotic issue and help improve patient management [10]. However, the lack of rapid, affordable, sensitive, and specific diagnostic tools is one of the main reasons for inefficient diagnosis.

PCR has revolutionized the field of infectious disease diagnosis. Multiplex PCR, a variant that uses several pairs of primers to amplify more than one target sequence in a single tube, was developed to detect different viruses in a single sample [11]. Previous studies showed that multiplex assays using a limited amount of sample had similar performance to that of traditional methods when used for clinical diagnosis [12]. Additionally, they have the benefit of detecting a large number of viruses in a short time.

The xTAG Respiratory Viral Panel Fast (RVP FAST) is a qualitative nucleic acid multiplex test that simultaneously detects and identifies the presence of nucleic acids from multiple respiratory viruses in a single tube. It works with samples such as nasopharyngeal swabs, nasal aspirates, nasopharyngeal aspirates, and bronchoalveolar lavages from individuals suspected of respiratory tract infections. The xTAG RVP FAST test has shown superior sensitivity when compared to single real-time PCR and conventional PCR assays [11, 13, 14]. In the present study, we used the xTAG RVP FAST assay to identify the viruses causing RI in children and the relationship between specific viruses and clinical outcome.

## 2. Materials and Methods

### 2.1. Patients

This cross-sectional study was held between November 2014 and June 2015. We enrolled 194 pediatric infants (2–24 months old) who had lower respiratory tract infections and were treated at the Vietnam National Children's Hospital. We collected nasopharyngeal aspirates from each participant. Patients were divided into 3 age groups. The first group included infants between 2 and 5 months (*n* = 71) who were breastfed and likely received passive immunity from their mothers. The second group included infants between 6 and 11 months old (*n* = 66) who spent most of their time at home. The third groups included infants between 12 and 24 months old (*n* = 57) who attended different nurseries.

### 2.2. Ethics Statement

This study was approved by the VNCH Ethics Committee (approval number: 521B/NCH-RICH). Written informed consent was obtained from the parents or legal guardians of the children enrolled in the study.

### 2.3. Clinical Specimens

We collected 2 ml of nasopharyngeal aspirate from each participant and stored the samples at −70°C until analysis. NPA collection was performed by trained nurses. The catheter was inserted into the nostril to a depth of 5 to 7 cm and drawn back while applying gentle suction with a syringe.

### 2.4. Nucleic Acid Extraction

NPA sample (200 *μ*l) from the patient was subjected to total nucleic extraction after addition of internal control bacteriophage MS2 (20 *μ*l) using the MagNA Pure LC Total Nucleic Acid Isolation kit (Roche Diagnostics, Germany) on Magna Pure LC 2.0 platform, following the manufacturer's instructions [15]. The method is based on magnetic-bead technology. The procedure included cellular destruction, nucleic acid binding on beads, and washing steps to remove cellular and purified nucleic acid elution. Extracted nucleic acids were eluted in 50 *µ*l of elution buffer and stored at −80°C for RVP FAST assay.

### 2.5. xTAG Respiratory Viral Panel Fast (RVP FAST)

RVP FAST assay detects influenza A and B (INFA, INFB) including subtypes H1N1 (1977), H1N1pdm09, H3N2, respiratory syncytial virus A and B (RSVA, RSVB), enteroviruses including rhinoviruses (EV/Rhi), human parainfluenza viruses 1–4 (PIV1-4), human metapneumovirus (hMPV), adenovirus (ADV), human coronavirus NL63 (hCoV NL63), hCoV HKU1, hCoV 229E, hCoV OC43, and human bocavirus (hBoV). The assay was performed according to the manufacturer's instructions. Assay performance was controlled using bacteriophage lambda included in every run. The assay comprised two steps: a multiplex PCR amplification step and hybridization step.

### 2.6. Multiplex RT-PCR

PCR amplification was performed on the Applied Biosystems Gene amp 9700 PCR (Applied Biosystems, USA) with the following program: preheating at 50°C for 20 min; template denaturation at 95°C for 15 min; 34 cycles of 95°C for 30 s, 59°C for 30 s, and 72°C for 30 s; and final extension at 72°C for 2 min and kept at 4°C until ready tousle.

### 2.7. Hybridization

The hybridization reaction was performed on Luminex 100/200 system (LMD, Toronto, Canada). The hybridization assay includes 20 *µ*l xTAG RVP FAST bead mix, 2 *µ*l amplified nucleic acid, and 75 *µ*l Streptavidin, R-Phycoerythrin Conjugate (SA-PE) reporter. All reagents were incubated at 45°C for 20 minutes. Signal acquisition was done on a Luminex 100/200 instrument. Data were analyzed and reported by xTAG Data Analysis Software (TDAS).

### 2.8. ARI Classification

The infections were classified as pneumonia, bronchiolitis, or bronchitis based on clinical findings and chest X-rays (CXR). Pneumonia was defined as ARI characterized by infiltrates on the CXR. Bronchiolitis was defined as ARI in children under 2 years who presented wheezing and hyperaeration and atelectasis or peribronchial thickening in the CXR. Bronchitis was defined as ARI in children over 2 years old who presented wheezing, hyperaeration, and peribronchial thickening on the CXR.

### 2.9. Statistical Analysis

Statistical analysis was performed using Fisher's exact test. *p* < 0.05 was considered significant (SPSS 13).

## 3. Results

### 3.1. Respiratory Virus Detection

The xTAG RVP FAST assay system detected one or more respiratory viruses in 73.7% (143/194) of the samples (Table 1). RSV was the most common virus (51%, 73/143), followed by enterovirus/rhinovirus (EV/Rhi), parainfluenza (PIV), adenovirus (AdV), influenza A/B, and human bocavirus (hBoV). Overall, 96 samples were positive for a single virus, 37 for 2 viruses, 9 for 3 viruses, and 1 for 4 viruses.

### 3.2. Viruses Causing Respiratory Infections in Different Age Groups

**Figure 1:**
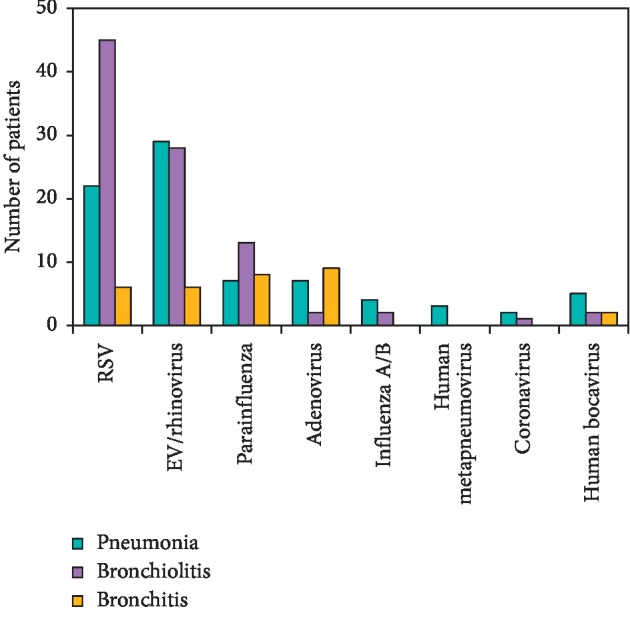
Bar graph showing the respiratory viruses detected in each age group. The most abundant viruses were respiratory syncytial virus (RSV) and enterovirus (EV)/rhinovirus (Rhi), which mainly infected patients in the 2–5 months group. ^*∗*^*p* < 0.001.

RSV and EV/Rhi were detected more frequently in children under 6 months (Figure 1). For other viruses, there was no difference among age groups.

### 3.3. Infection with Respiratory Viruses Depends on Inflammation of the Respiratory Tract

Children with viral infection in the upper respiratory tract showed symptoms such as runny nose, cough, and hoarseness. Some of them also present lower respiratory tract symptoms such as wheezing, severe cough, breathlessness, and respiratory distress, which may be due to bronchiolitis or pneumonia.

**Figure 2:**
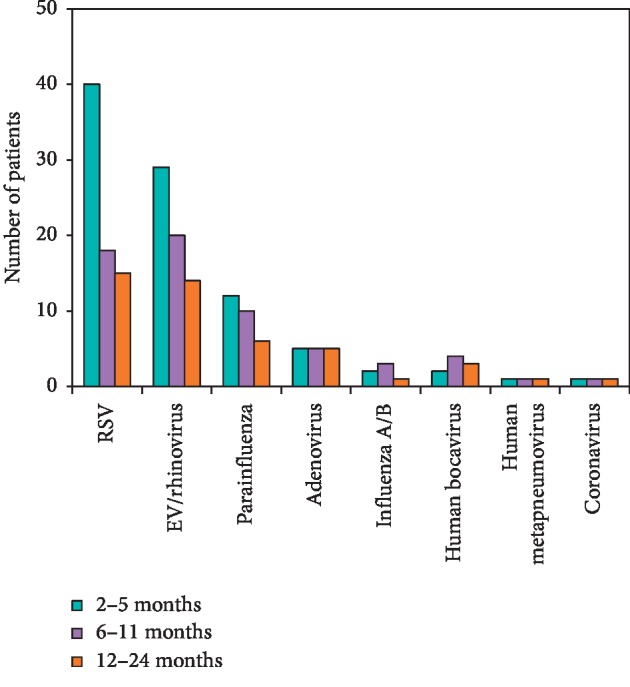
Bar graph illustrating the association between specific viral infections and clinical manifestations of acute respiratory infections. In the respiratory syncytial virus- (RSV-) infected group, the prevalence of pneumonia symptoms was significantly lower than that of bronchiolitis symptoms and significantly higher than that of bronchitis symptoms. In the enterovirus (EV)/rhinovirus (Rhi) infected group, the prevalence of pneumonia symptoms was significantly higher than that of bronchitis symptoms. Other viruses showed a similar prevalence of each manifestation. ^*∗*^*p* < 0.05.

We divided the patients according to three clinical manifestations: pneumonia, bronchiolitis, and bronchitis, and investigated whether the detected viruses were associated with a specific clinical manifestation. Our analysis showed that in most cases, RSV infections induced bronchiolitis (*n* = 45), followed by pneumonia (*n* = 22, *p*=0.004) and bronchitis (*n* = 6, *p*=0.0015). EV/Rhi infections more often induced pneumonia or bronchiolitis (*n* = 19 for both) instead of bronchitis (*n* = 6, *p*=0.03) (Figure 2). Other viruses showed a similar prevalence of each clinical manifestation.

### 3.4. Clinical Respiratory Signs of Patients with Respiratory Virus Infections

All respiratory viruses may cause symptoms such as nasal congestion, runny nose, wheezing, and cough. We found no significant association between the viruses and a specific symptom.

### 3.5. Clinical Data in Patients with Respiratory Viral Infection

**Figure 3:**
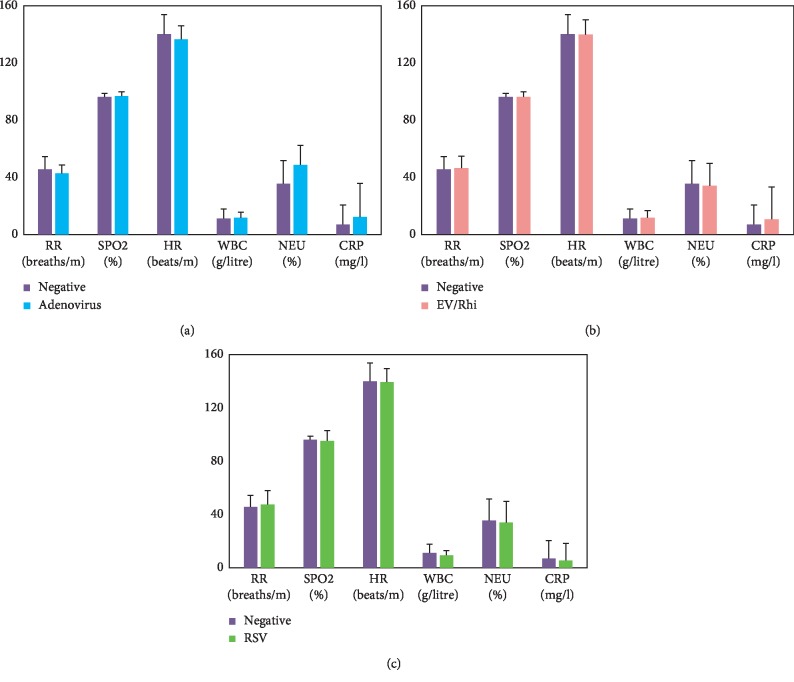
Bar graphs showing the association between specific viral infection and subclinical and clinical signs. (a) Comparison of clinical data between adenovirus infection and the negative group. (b) Comparison between enterovirus/rhinovirus infection and the negative group. (c) Comparison between respiratory syncytial virus (RSV) infection and the negative group. RR: respiratory rate; SPO_2_: peripheral capillary oxygen saturation; HR: heart rate; WBC: white blood cell count; NEU: neutrophil count; CRP: C-reactive protein.

We studied whether the presence of specific viral agents was associated with clinical parameters such as respiratory rate (breaths/min), heart rate (beats/min), and peripheral capillary oxygen saturation (SPO_2_, %) or with blood test parameters such as white blood count, neutrophil count, or C-reactive protein (CRP). Elevated neutrophils were associated with adenovirus infection (OR = 1.4, *p*=0.006, Figure 3). There were no other noticeable associations.

CRP was slightly higher in the adenovirus and enterovirus/rhinovirus infection groups than in the negative group (*p*=0.29 and 0.32, respectively). Neutrophils were significantly higher in the adenovirus infection group (48.7%) than in the negative group (35.6%, *p*=0.0058).

## 4. Discussion

In this study, we calculated the frequency of different respiratory viruses present in children with lower respiratory tract infection at the Vietnam National Children's Hospital. The high proportion of viruses detected in pediatric ARI patients agreed with previous studies done in Vietnam [5]. Tran et al. found that in the South of Vietnam, human rhinovirus (HRV) and human bocavirus were associated with the severity of children with respiratory infections, while those viruses were detected rarely in the North of Vietnam due to the difference of the location and climate between the South and North of Vietnam. [16]. RSV and EV/Rhi were the most frequent viruses, which was similar to the findings of a study done in Southern Vietnam [6].

RSV, rhinoviruses, influenza viruses, parainfluenza viruses, enteroviruses, coronaviruses, and certain strains of adenovirus are the leading causes of viral respiratory infections in children. The nasal or respiratory secretions from children with viral respiratory tract infections contain more viruses than those from infected adults. The increased output of viruses, together with an overall reduced attention to hygiene, makes children more likely to spread their infection to others.

When viruses invade the cells of the respiratory tract, they trigger inflammation and mucus production, which causes nasal congestion, runny nose, scratchy throat, and cough. The small airways of young children can be significantly narrowed by inflammation and mucus, making breathing difficult. Airway problems are most common in infections caused by parainfluenza viruses, RSV, and human metapneumovirus [1, 8, 17]. Bronchiolitis occurs predominantly in the first year of life and with decreasing frequency in the second and third years. It is characterized by inflammatory obstruction of the small airways and hyperinflation of the lungs and typically presents along with breathing problems and wheezing. RSV is the primary causative agent of bronchiolitis worldwide, causing between 70 or 80 percent of ARIs during the high season [18–20]. RSV was the only virus associated with bronchiolitis in this study.

Neutrophils are immune cells that are present in many lung diseases associated with acute respiratory distress syndrome (ARDS) and may contribute to acute lung injury. Neutrophils are poorly studied with respect to viral infection. We observed an association between elevated neutrophil count and adenovirus infection, which might indicate an association between neutrophil count and damage to the alveolar epithelium.

Finding biomarkers to diagnose specific viral infections is essential to improve patient care [21]. CRP is an acute phase protein synthesized by the liver in response to IL-6 increase, which is used as a biomarker of inflammation [22] and to distinguish between bacterial and viral infections. It is not well known if CRP levels differ between different viral respiratory infections. In this study, we found no significant association between CRP and a specific virus.

## 5. Conclusions

Bronchiolitis was the most common clinical characteristic of lower respiratory tract infection at the Vietnam National Children's Hospital.

xTAG RVP FAST can effectively detect different virus from specimens with low viral loads. The assay should be applied in the clinic for the screening of multiple respiratory viral infections.

## Figures and Tables

**Table 1:** Viruses detected in 194 patients with acute respiratory infections using the respiratory viral panel Fast assay.

	Total	RSV	Enterovirus/rhinovirus	Parainfluenza	Adenovirus	Influenza A/B	Human bocavirus	Human metapneumovirus	Coronavirus
Total positive specimens	143	73	62	28	15	6	9	3	3
Single infection	96	44	28	12	3	3	3	1	1
Double infection	37	22	26	11	7	3	2	1	1
Triple infection	9	6	7	4	5	0	3	1	1
Fourth infection	1	1	1	1	0	0	1	0	0

## Data Availability

The data used to support the findings of this study are included within the article.
